# Relevance of inducible nitric oxide synthase for immune control of *Mycobacterium avium* subspecies *paratuberculosis* infection in mice

**DOI:** 10.1080/21505594.2020.1763055

**Published:** 2020-05-14

**Authors:** Ketema Abdissa, Nanthapon Ruangkiattikul, Wiebke Ahrend, Andreas Nerlich, Andreas Beineke, Kristin Laarmann, Nina Janze, Ulrike Lobermeyer, Abdulhadi Suwandi, Christine Falk, Ulrike Schleicher, Christian Bogdan, Siegfried Weiss, Ralph Goethe

**Affiliations:** a Institute for Microbiology, University of Veterinary Medicine Hannover, Hannover, Germany; b Department of Molecular Immunology, Helmholtz Centre for Infection Research, Braunschweig, Germany; c Institute for Pathology, University of Veterinary Medicine Hannover, Hannover, Germany; d Mouse Pathology, Helmholtz Centre for Infection Research, Braunschweig, Germany; e Institute of Transplant Immunology, Hannover Medical School, Hannover, Germany; f Mikrobiologisches Institut, Klinische Mikrobiologie, Immunologie Und Hygiene, Friedrich-Alexander-Universität (FAU) Erlangen-Nürnberg and Universitätsklinikum Erlangen, Erlangen, Germany; g Institute of Immunology, Hannover Medical School, Hannover, Germany

**Keywords:** Mycobacterium, macrophage, inducible or type 2 nitric oxide synthase, paratuberculosis, Johne’s disease

## Abstract

Mycobacterium avium

subspecies *paratuberculosis* (MAP) causes Johne’s disease (JD), an incurable chronic intestinal bowel disease in ruminants. JD occurs worldwide and causes enormous economic burden in dairy industry. Research on JD pathobiology is hampered by its complexity which cannot completely be mimicked by small animal models. As a model the mouse allows dissecting some pathogenicity features of MAP. However, for unknown reasons MAP exhibits reduced growth in granulomas of infected mice compared to other *Mycobacterium avium* subspecies. Here, we characterized immune reactions of MAP-infected C57BL/6 mice. After infection, mice appeared fully immunocompetent. A strong antigen-specific T cell response was elicited indicated by IFNγ production of splenic T cells re-stimulated with MAP antigens. Function of splenic dendritic cells and proliferation of adoptively transferred antigen-specific CD4^+^ T cells was unaltered. Isolated splenic myeloid cells from infected mice revealed that MAP resides in CD11b^+^ macrophages. Importantly, sorted CD11b^+^CD11c^−^ cells expressed high level of type 2 nitric oxide synthase (NOS2) but only low levels of pro- and anti-inflammatory cytokines. Correspondingly, MAP-infected MAC2 expressing myeloid cells in spleen and liver granuloma displayed strong expression of NOS2. In livers of infected *Nos2^−/−^*mice higher bacterial loads, more granuloma and larger areas of tissue damage were observed 5 weeks post infection compared to wild type mice. *In vitro*, MAP was sensitive to NO released by a NO-donor. Thus, a strong T cell response and concomitant NOS2/NO activity appears to control MAP infection, but allows development of chronicity and pathogen persistence. A similar mechanism might explain persistence of MAP in ruminants.

## Introduction

Johne’s disease (JD) or paratuberculosis is a chronic intestinal infection of ruminants caused by *Mycobacterium avium* subspecies *paratuberculosis* (MAP). The disease causes severe economic burden for cattle farming worldwide [1–3]. In addition, MAP is suspected to have anthropozoonotic potential [4–7]. MAP is proposed to initiate or exacerbate Crohn’s disease (CD) and autoimmune diseases in humans [8–12]. Along this hypothesis, experimental evidence has accumulated that MAP is an exacerbating factor of inflammatory bowel disease (IBD) [13,14].

MAP is a pathogenic subspecies of *M. avium* and genetically closely related to *M. avium* subsp. *avium* (MAA) and *M. avium* subsp. *hominissuis* (MAH) [15]. Little is known about the mechanisms by which MAP contribute to pathology of JD. This is mainly due to the difficult biology of the disease and the unique features of the pathogen. In contrast to MAA and MAH, MAP grows extremely slowly in culture and genetic manipulation is difficult [16].

MAP is usually transmitted via the fecal–oral route from adults to neonatal calves. *In vivo*, it invades subepithelial macrophages and persists within these cells. Progression of disease is variable. Infection may either be cleared in some animals or developing into subclinical disease which might progress to clinical JD after several years. Clinical signs are persistent diarrhea and emaciation. However, some infected animals may remain in the subclinical state and never develop clinical symptoms [17]. Severity of the clinical disease does not always correlate with the number of macrophages infected by MAP or the extent of intestinal inflammation. Accordingly, clinical signs can be observed in animals with multibacillar JD, i.e. high numbers of MAP-infected macrophages in the inflamed intestinal tissue, as well as in animals with paucibacillar JD, i.e. with low numbers of MAP-infected macrophages in the affected intestine. The reasons for the different pathological responses seem to depend on host immune competence and the type of immune response elicited [18].

Cellular and molecular immunopathogenesis of JD is incompletely understood. Following oral uptake, MAP enters deeper intestinal tissue most likely via M cells of the distal ileum [19,20]. An entry via goblet cells is discussed [21,22]. After breaching the epithelial barrier, MAP is engulfed by subepithelial macrophages and spread to the draining lymph nodes [23]. The following steps of pathogenesis are still elusive. Consensus consists that the ability of MAP to persist and/or to replicate in infected gut macrophages is an essential prerequisite for ongoing infection. Decisive for this appears the IFNγ production of MAP specific CD4^+^ T cells. It activates macrophages to efficiently kill MAP. Indeed infected animals develop an early cellular immune response which however is lost during the course of infection and replaced by a humoral response [23]. Often, the cellular immune response is unable to control infection while it is facilitating progressive granulomatous inflammation of the gut. In addition, the extent of lesions as well as the course of disease progression might indicate the quality of the cellular immune response against MAP [23]. Nevertheless, factors other than IFNγ might contribute to the control of MAP replication. Similarly, what regulates the extent granulomatous inflammation is not well defined.

Species specificity and the peculiarity of pathogenesis of JD largely restrict the use of laboratory animals for MAP research. However, even if it is not possible to mirror the complete picture of JD in laboratory animals they may help to understand certain aspects of MAP pathogenicity and disease. Since histological features such as granulomatous inflammation and immunological responses in mice are broadly similar to those in ruminants [24], mouse models should help to understand aspects of host-pathogen interactions and to identify vaccine candidates [24].

Usually, mice are infected intraperitoneally. Oral infection is less reproducible and does not reproduce the typical intestinal disease of JD in cattle [24–26]. Despite of a multitude of MAP studies in mice, immunopathology of MAP-infected mice was not systematically analyzed thus far. Nevertheless, a common observation was that MAP is able to multiply only poorly or not at all. This is in striking contrast to infection of mice with *Mycobacterium avium* subspecies MAA and MAH [27,28]. On the other hand, mice are not able to clear MAP even after months of persistence. These features are reminiscent of *Mycobacterium tuberculosis* (MTB) infection of mice.

In ruminants as well as in mice, the ability of MAP to infect and survive in host macrophages is thought to be a crucial property to establish an infection and for disease progression [29–31]. Accordingly, MAP is able to inhibit the phagosomal maturation of the infected macrophage and prevent intracellular killing [32]. As consequence, persistent granulomatous inflammation and granuloma formation are observed in MAP-infected hosts [8,33]. In mice, MAP persistence appears to be the result of an inadequate immune reaction against the bacteria. For instance, we recently reported the absence of type I interferon induction which contributed to the survival of MAP in mice [34]. Nevertheless, the various host factors affecting growth of MAP in such animals are far from being understood.

In the present study, we characterized MAP-specific immune reactions in C57BL/6J mice. Our data revealed that mice exhibit a specific cellular immune response. Granulomas were formed in spleen and liver. Bacteria resided in NOS2 expressing myeloid cells of liver and spleen granuloma. Expression of NOS2 and production of NO were found to restrict growth of MAP and progressive, granulomatous inflammation. This indicates that NOS2 plays a decisive role in the control of MAP infection in mice.

## Materials and methods

### Mice

Female C57BL/6J mice were purchased from Janvier (Le Genest-Saint-Isle, France) and maintained under specific pathogenic-free conditions (SPF) at the animal facility of the Helmholtz Centre for Infection Research (HZI), Braunschweig, Germany. *Thy1.1* OVA albumin transgenic II (OT-II) C57B6L/J mice were bred at HZI. *Nos2*^−/−^ on a C57BL/6J background were originally obtained from the Jackson Laboratories (Bar Harbor, ME, USA; stock no. 2609) [35] and bred and maintained under specific pathogen-free conditions at the Franz Penzoldt Preclinical Animal Research Center of FAU and Universitätsklinikum Erlangen. Mice were infected at the age of 7–12 weeks. In all experiments, mice could feed ad-libitum and had unlimited access to water. Approval of study was granted from research ethics committee of the local authority LAVES in Lower Saxony (permission No. 3392 42502-04-13/1192).

### Culture of MAP and infection

MAP6783 (DSM 44135) was grown in Middlebrook 7H9 broth (BD) supplemented with 2 mg/L Mycobactin J (IDVet), 0.5% glycerol and 10% OADC. To attain early logarithmic phase of growth, initial inoculum of optical density 600 (OD_600nm_) 0.2 was grown at 37°C under stirring conditions (130 rpm) until a final OD of ~1. The bacterial culture was washed 3 times with Dulbecco’s phosphate buffered saline (DPBS). To avoid clumping, bacterial suspension was briefly vortexed with 3 mm glass beads. Bacterial suspension was adjusted to OD_600_ of 5 in DPBS. Mice were infected intraperitoneally (i.p) with 200 µL (~10^8^ CFU) of bacteria and followed for up to 5 weeks.

### Organ sampling and plating

Liver were collected aseptically and weighed. For plating, ~300 mg of liver sections were homogenized in 1 mL sterile PBS containing 0.1% Triton-X100 in the presence of sterile 3 mm glass beads by beating three times for 20 s with 5 min cooling intervals using Hybaid Ribolyser. Serial dilutions of homogenous samples were plated on Middlebrook 7H10 agar containing 2 mg/L Mycobactin J and 10% OADC. Colonies were counted after 5–8 weeks. The bacterial load was calculated as CFU/organ.

### Preparation of mycobacterial lysate

MAP cells grown in Watson Reid medium were pelleted, washed twice with 20 mL Tris-HCl (pH 7.5), and resuspended in 50 mM Tris-HCl (pH 8.0). Then cells were passed through French press (4 cycles), with 14,000 PSI with intermediate cooling on ice. Lysates were centrifuged for 30 min at 12,000 rpm at 4°C to remove cell debris. Subsequently, concentration of proteins in the supernatant was analyzed by MicroBCA (Micro BCAssay UP75860 Protein Quantification Kit (Uptima) and quantified with an Epoch microplate reader (BioTek).

### Flow cytometry and cell sorting

Spleen cell suspensions were prepared by gently flushing the organs with Iscove’s complete medium (IMDM) supplemented with 10% heat inactivated fetal calf serum, penicillin 100 unit/mL, streptomycin 100 µg/mL, 2 mM L-glutamine, 50 µM 2-mercaptoethanol. Then, cells were filtered through 70 µm and finally through 50 µm diameter cell strainers. Red blood cells were removed by erythrocyte lysis buffer (14.2 mM sodium hydrogen carbonate [NaHCO_3_], 155 mM ammonium chloride [NH_4_Cl], 0.1 mM EDTA, at final pH of 7.3). Cells were stained in FACS buffer (PBS containing 2 mM EDTA, 2% FBS). Anti-Gr-1 (clone RB6.8C5) and anti-Ly6C (clone AL-21) were purchased from BD Bioscience. Other antibodies were purchased from eBioscience: Anti-CD16/32 (clone 2.4G2, FCR block), anti-CD11b (clone M1/70), anti-CD11c (clone N418), anti-MHC-II I-A/I-E (clone M5/114.15.2), anti-CD86 (clone GL1), anti-CD3 (clone 17A2), anti-CD4 (clone RMA4.5), anti-CD90.1 (clone HIS51), and anti-NOS2 (clone CXFNT). Anti-MAC-2 (clone M3/38) was purchased from Biolegend. Data were acquired on a LSR II analyzer (BD, NJ, USA). Data analysis was done using FACSDiva software (BD) or FlowJo (TreeStar). Cell sorting was done on BD FACSAria-II. Re-analysis of sorted cells was done for purity confirmation.

### Ex vivo *antigen dependent T cell proliferation*

Splenic conventional dendritic cells, CD11c^hi^CD11b^±^ (cDC) were sorted and pulsed with Endograde OVA protein (Hyglos, 100 µg/mL) or OVA peptide (Aa_323–339,_ 1 µg/mL) for 1 h at 37°C in IMDM complete medium. CD4^+^ T cells were isolated from the spleen of OT-II mice using Dynabeads® Untouched™ Mouse CD4^+^ Cells Kit (Invitrogen) with purity of ≥90%. CD4^+^ cells were stained with 5 µM carboxyfluorescein diacetate succinimidyl ester (CFSE, Invitrogen) and incubated for 10 min at 37^°^C and washed three times in complete medium. Finally, viable 3 × 10^4^ DC were co-cultured with viable 3 × 10^5^ CD4^+^ T cells in complete medium. Proliferation was measured after 3 days for peptide or 4 days for protein.

### Antigen-specific CD4^+^ T cell stimulation assay

To generate dendritic cells *in vitro*, C57B6/J bone marrow cells were cultured at a density of 5 × 10^6^ cells in 10 cm dishes in 10 mL complete IMDM medium supplemented with 20% conditioned medium of X-63 cells producing GMCSF and 20 ng/mL murine IL-4. On day 3, new 10 mL complete medium containing growth factors was added to the cultures. On day 6, non-adherent cells were seeded overnight in 96 well plates at density of 5 × 10^4^ cells per well in the presence of 20 ng/mL *E. coli* LPS. The next day, cells were co-cultured with CD4^+^ T cells isolated from MAP-infected mice or PBS controls in the presence 50 µg/mL MAP lysate. CD4^+^ T cells cultured in the presence of plate bounding anti-CD3 (5 µg/mL, clone 45-2c11) and anti-CD28 (5 µg/mL, clone 37.51) were used as positive control. On day 5 after co-culture, the supernatants were collected and IFNγ was measured using ELISA.

### In vivo *T cell proliferation*

Naïve CFSE-labeled *Thy1.1* expressing OT-II CD4^+^ T cells (2 × 10^6^) were injected intravenously into infected or control mice. After 24 h, 200 µg OVA protein was administered i.p. Three days after, mice were sacrificed and spleens were collected. *In vivo* proliferation of *Th1.1* expressing CD4^+^ T cells was monitored by CFSE dilution using flow cytometry.

### In vitro *infection of bone marrow derived macrophages*

Mice were sacrificed after CO_2_ asphyxiation by cervical dislocation. Femur bones were collected aseptically. Bones were flushed with PBS and filtered in 50 µm filter and centrifuged at 1000 RPM for 5 min. To get rid off red blood cells, cells were resuspended in RBC lysis buffer and collected after centrifugation. Cells were resuspended in a freezing medium (fetal calf serum containing 10% DMSO) and stored in liquid nitrogen until use. WT and Nos2^−/−^ C57B6/J bone marrow cells were cultured at a density of 5 × 10^6^ in DMEM complete medium (DMEM supplemented with 10% heat inactivated fetal calf serum, 100 units/mL penicillin, 100 µg/mL streptomycin, 2 mM L-glutamine) containing 20% L929 cell conditioned medium containing M-CSF (macrophage colony-stimulating factor). Medium was changed on day 3 and cells were allowed to grow for 7 more days. On day 10, ~2 × 10^6^ cells were seeded in six well plates (Sarstedt) and incubated overnight. Cells were then infected with MAP at a MOI ~10 in complete medium without antibiotics. In some conditions, cells were treated with 100 ng/mL recombinant murine IFNγ (Miltenyi Biotec) throughout the experiment. Medium was changed to complete medium with antibiotics and changed every day until day 7.

### Quantitative real time PCR

After sorting spleen cells from MAP-infected mice or PBS control, cells were kept in 500 µL DNA/RNA Shield™ (Zymo Research). Afterward, RNA extraction was carried out using Direct-zol^TM^ RNA Miniprep kit (Zymo Research). RNA from bone marrow derived macrophages was purified using RNeasy purification kit (Qiagen). RNA was reverse transcribed using M-MLV transcriptase (Promega) and oligo-(dT)_12–18_ primers (Carl Roth). Quantitation of expression of selected genes was done using TaqMan® Gene Expression Assays (Applied Biosystems) (assay ID; IFNγ: Mm01168134_m1, IL-10: Mm00439616_m1, IL-6: Mm00446190_m1). Primers used for SYBR based expression assays were *Tnf*: for_ATGAGCACAGAAAGCATGATC, rev_TACAGGCTTGTCACTCGAATT; *Il1b*: for_TTGACGGACCCCAAAAGATG, rev_AGAAGGTGCTCATGTCCTCA; *Nos2*: for_CCCAGCACAAAGGGCTCAAA, rev_GCACCTGGAACAGCACTCTC; *Arg1*: for_GATGTCCCTAATGACAGCTCC, rev_AGCACCACACTGACTCTTCC; *Rps*9: for_CTGGACGAGGGCAAGATGAAGC, rev_TGACGTTGGCGGATGAGCACA. Fold induction was calculated using housekeeping gene *Rps9* as standard as described [34].

### Quantification of intracellular bacteria by PCR

Whole cell (eukaryotic and bacterial) DNA was extracted from sorted, 3% paraformaldehyde fixed cells or from macrophages infected with MAP *in vitro*. Briefly, pelleted cells were re-suspended in 500 µL lysis buffer (TE buffer with 1% SDS), zirconium beads were added and cells were disrupted using a tissue homogenizer. The homogenate was sonicated using Branson sonifier 450. Supernatant was collected after centrifugation. After adding an equal volume of TE buffer, RNA was removed by adding 20 µL RNase A (Roche) followed by a 1 h incubation at 37^°^C. To reverse the paraformaldehyde cross link, 30 µL of 4 M NaCl was added to fixed samples and incubated for 5 h at 65^°^C. DNA was extracted using standard phenol-chloroform extraction method. Bacterial DNA was determined by PCR using the MAP-DNA specific primers and normalized against eukaryotic *Cxcl2* (*Mip2a*) promoter. Primers sequences used were: MAP-DNA: for_CTCGACCGCTAATTGAGAG, rev_CACAACCACCTCCGTAACC; *Mip2a*: for_GAAGGGCAGGGCAGTAGAAT, rev_ATGGCGCTAGGCTGAAGTG.

### Histopathology

Upon isolation, tissues were fixed with 4% (v/v) formalin and embedded in paraffin. Approximately 3 µm thick sections were cut and stained with hematoxylin/eosin according to standard laboratory procedures. For Immunofluorescence staining, paraffin embedded sections were deparaffinized in 100% xylol and gradient ethanol concentrations. Antigen unmasking step was performed by pressure cooking of the deparaffinized section in 10 mM citrate buffer (pH = 6). Staining procedure was performed using the following primary antibodies: rat anti-mouse NOS2 (eBioscience, 1:200 dilution), rat anti-mouse MAC-2 (Biolegend, 1:200 dilution), and self-produced anti-MAP heparin binding hemagglutinin (HBHA, 1:200 dilution). Secondary antibodies: goat anti-rat Alexa Fluor 488 and anti-rabbit Alexa Fluor 568 were used at a final dilution of 1:500.

### Confocal microscopy

Confocal microscopy was performed with an inverted Leica TCS SP5 microscope, equipped with lasers for 405, 488, and 561 nm excitation. Images were acquired with a 63×/1.4 NA HCX PL APO objective and image pixel size of 240 nm. The following settings were used for detection: DAPI: 410–480 nm, AlexaFluor-488: 489–560 nm, AlexaFluor-568: 569–640 nm. Image stacks with a *z*-distance of 0.5 μm per plane were acquired using a 1-Airy-unit pinhole diameter in sequential imaging mode to avoid bleed through. Images shown in Figure 3 were acquired using a LSM 780 confocal laser-scanning microscope with a 40×/1.3 Plan-Apochromat objective controlled by Zen 2012 (Carl Zeiss Microscopy GmbH) in spectral imaging mode. Spectral image stacks were acquired in λ mode using 4 laser lines (405, 488, 561, and 633 nm) and the QUASAR detector (detection range 411–695 nm) with a z-distance of 0.5 μm per plane and the pinhole set to 1-Airy-unit. λ stacks were subsequently linear unmixed with Zen 2012 (Carl Zeiss Microscopy GmbH). Maximum intensity projections were calculated for display purposes and brightness and contrast were adjusted identically for all images of the same dataset using ImageJ/Fiji.

### IFNγ ELISA

The concentration of IFNγ in supernatant of co-cultured dendritic cells and T cells co-culture was analyzed using coating (clone A.N 18) and detection (clone R46A2) antibodies. In brief, coating rat anti-mouse IFNγ was incubated in 50 µL coating buffer in 96 well plates (MaxiSorb TM Immunoplates, Nunc) over night. The 96 well plates were then blocked for 1 h with 3% BSA in 0.05% Tween 20. Diluted sera were added to the wells and incubated for 2 h at room temperature. Biotinylated detection rat anti-mouse IFNγ was added and incubated for 1 h. Then horseradish peroxidase (HRP) conjugated streptavidin (BD) was added and incubated for 30 min and the bound HRP was detected with o-phenylenediamine (OPD) substrate in terms of absorbance at 490 nm using ELISA reader XFluor software (Tecan SUNRISE).

### Multiplex ELISA

Blood was collected via cardiac puncture in 500 Serum-Gel tubes (Sarstedt). Serum was separated by centrifugation at 10,000 rpm for 4 min at room temperature and kept at −80°C until analysis. Serum concentration of cytokines and chemokines were quantified by LUMINEX based mouse cytokine 23-plex assay following manufacturer’s instruction (Bio-Rad, USA).

### In vitro *GSNO susceptibly assay*

About 10^4^ bacterial cells (*M. smegmatis* mc^2^155, *M. avium*25291 and MAP6783) from early exponential growth phase were incubated in 100 µL PBS containing 4 and 8 mM concentration of GNSO for 4 and 8 h. As control, equal numbers of bacterial cells were incubated with PBS. After 4 and 8 h of incubation, serial dilutions of bacteria were plated on Middlebrook agar plates and bacterial survival was monitored by CFU counting. To determine the amount of nitrite, as an indicator of NO released from the respective concentration of GSNO, the culture supernatants were collected at 0, 1, 2, 4, 6, and 8 h of incubation with the respective bacterial strains.

### Nitrite assay

To quantify the amount of nitric oxide (NO) after *in vitro* MAP infection and *in vitro* GSNO assay, nitrite content in the cell culture supernatant was measured using the Griess reaction as described by the manufacturer (Promega).

### Statistical analysis

Data were analyzed with the GraphPad Prism version 5 software. Mean ± standard error of the mean (mean ± SEM) was used for data description. Statistical test between two groups was determined using Student’s *t-*test. Difference between more than two groups was determined either with one way or two-way analysis of variance (ANOVA) using Dunnett’s multiple comparisons test or Bonferroni’s multiple comparison tests. Cut off p-value of <0.05 was considered as statistically significant difference (*p < 0.05, **p < 0.01, ***p < 0.005).

## Results

### Persistence of MAP in liver granuloma-associated macrophages in mice

In mice, MAP is able to persist for extended periods of time in spleen, liver, mesentery, and intestine after intraperitoneal application [14,34]. To understand the mechanisms that account for pathogen persistence, we infected 8 weeks old female C57B6/J mice intraperitoneally with a high dose of 10^8^ CFU MAP.

**Figure 1.**
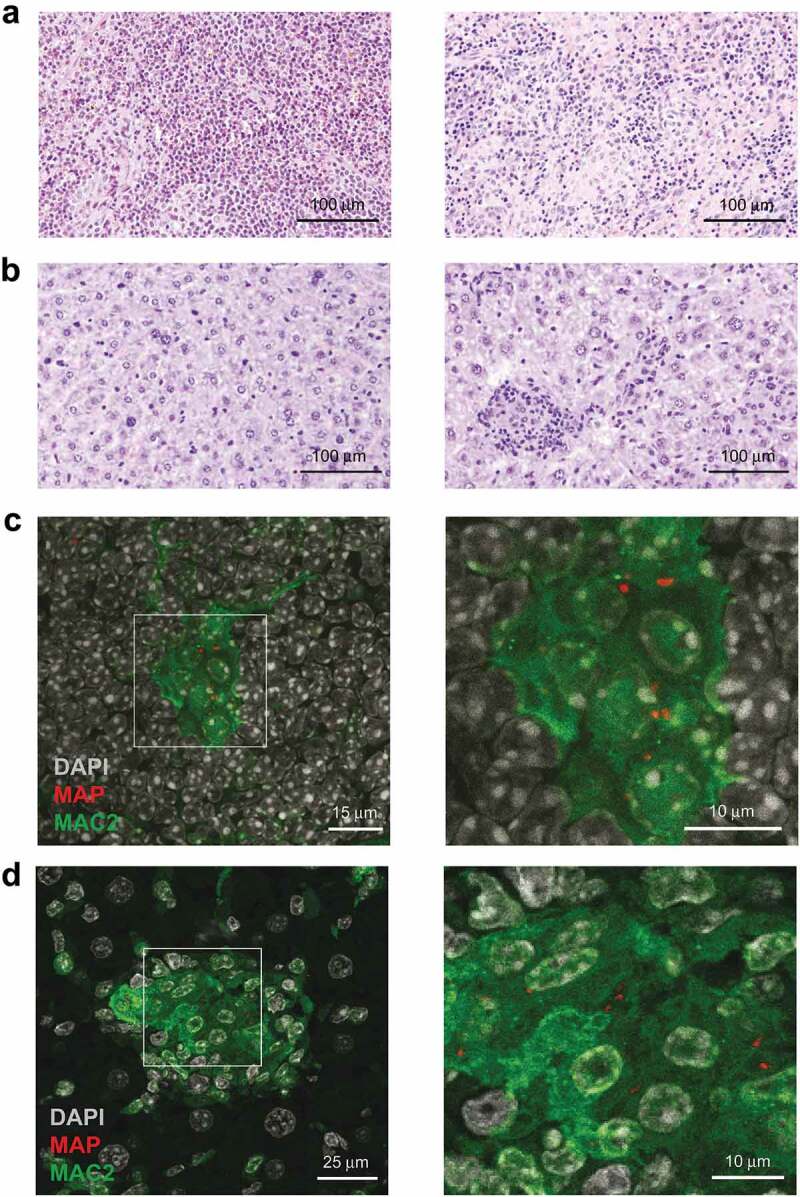
Histopathology of spleen and liver in MAP-infected mice.

Mice infected for 5 weeks exhibited multifocal granulomatous splenitis with accumulation of irregular shaped and sized randomly distributed clusters of epithelioid macrophages surrounded by lymphocytes (Figure 1(a)). In livers granuloma of different shapes and sizes with no evidence of caseation or necrosis were observed (Figure 1(b)). Immuno-histochemical analysis of spleens (Figure 1(c)) and livers (Figure 1(d)) of infected mice demonstrated that in both organs MAP resided in MAC2 (galectin-3) expressing macrophages. In agreement, we found few acid-fast bacilli in well-formed granuloma after Ziehl-Neelsen staining of livers of infected mice (Figure S1A). As shown in Figures S1B and S2A (upper panels), histology revealed no inflammatory lesions in the liver at 1 day post infection. At 2 weeks, individual focal accumulations of macrophages and single neutrophilic granulocytes were present in the parenchyma. At 3 weeks, we observed increased numbers of activated macrophages with an epithelioid morphology (epithelioid macrophages) and lymphocytes, characteristic for granuloma formation in infected mice. Together, these results demonstrate the central role of macrophages for pathology in MAP-infected mice.

### MAP infection does not impair dendritic cell function or T cell responses

**Figure 2.**
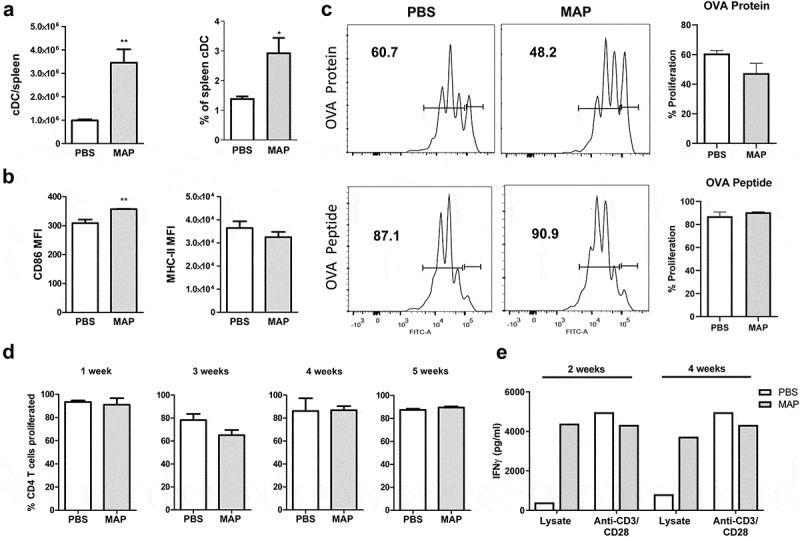
MAP infection of mice does not impair dendritic cell function and T cell response.

We have recently shown that MAA subverts the immune response of the host by influencing the activity of antigen-presenting cells as well as T cells [27]. To see whether persistence of MAP is also associated with an impaired cellular immune response, we first characterized conventional dendritic cells (cDC, CD11c^hi^CD11b^±^) from spleens of MAP-infected mice. Five weeks post infection the number of cDC had significantly increased (Figure 2(a)). The expression levels of MHC class II of such cells were comparable to the control group while expression of the co-stimulator CD86 was slightly enhanced (Figure 2(b)).

To prove their functional capacity, sorted cDC from spleens of mice infected with MAP for 4 weeks were sensitized with OVA protein or OVA peptide (Aa_323-339_) and co-cultured with OT-II T cells to monitor their antigen-processing and antigen-presentation capacity. No difference in the capacity of cDC to stimulate T cell proliferation was observed after sensitizing cDC from infected or control mice with OVA peptide. Nearly all OT-II cells proliferated (Figure 2(c)). Stimulatory capacity of cDC from infected mice was only slightly lower compared to controls when OVA protein was used for sensitization (Figure 2(c)). Thus, the capacity of cDC to process and present antigens appears to remain intact even after MAP infection.

As *in vitro* stimulation experiments inadequately reflect the complexity of host-pathogen interactions *in vivo*, we additionally tested T cell proliferation in infected mice. To this end, we adoptively transferred CFSE-labeled splenic OT-II T cells into uninfected mice or mice infected with MAP for 1, 3, 4, or 5 weeks. OVA protein was administered after 24 h and proliferation of transferred OT-II T cells was monitored by flow cytometry after additional 3 days. As shown in Figure 2(d), independent of the duration of infection, *in vivo* T cell proliferation was identical in MAP-infected and uninfected control mice. This suggests that despite the ongoing MAP infection, T cell immune response remained intact.

To determine whether a MAP-specific CD4^+^ T cell response was successfully initiated following infection, 14 and 30 days after MAP infection, CD4^+^ T cells were isolated from pooled splenocytes. These CD4^+^ T cells were co-cultured with bone marrow-derived dendritic cells in the presence of 50 µg/mL MAP lysate for 5 days. Upon re-stimulation with the MAP antigens, higher amounts of IFNγ were secreted by CD4^+^ T cell from MAP-infected mice compared to T cells from uninfected animals. In contrast, the release of IFNγ by both groups of CD4^+^ T cells was similar following polyclonal stimulation of CD4^+^ T cells with anti-CD3 and anti-CD28 antibody (Figure 2(e)). This indicates that upon infection a strong MAP-specific T cell response is induced.

### CD11b expressing myeloid cells exhibit enhanced type 2 nitric oxide synthase expression upon MAP infection

**Figure 3.**
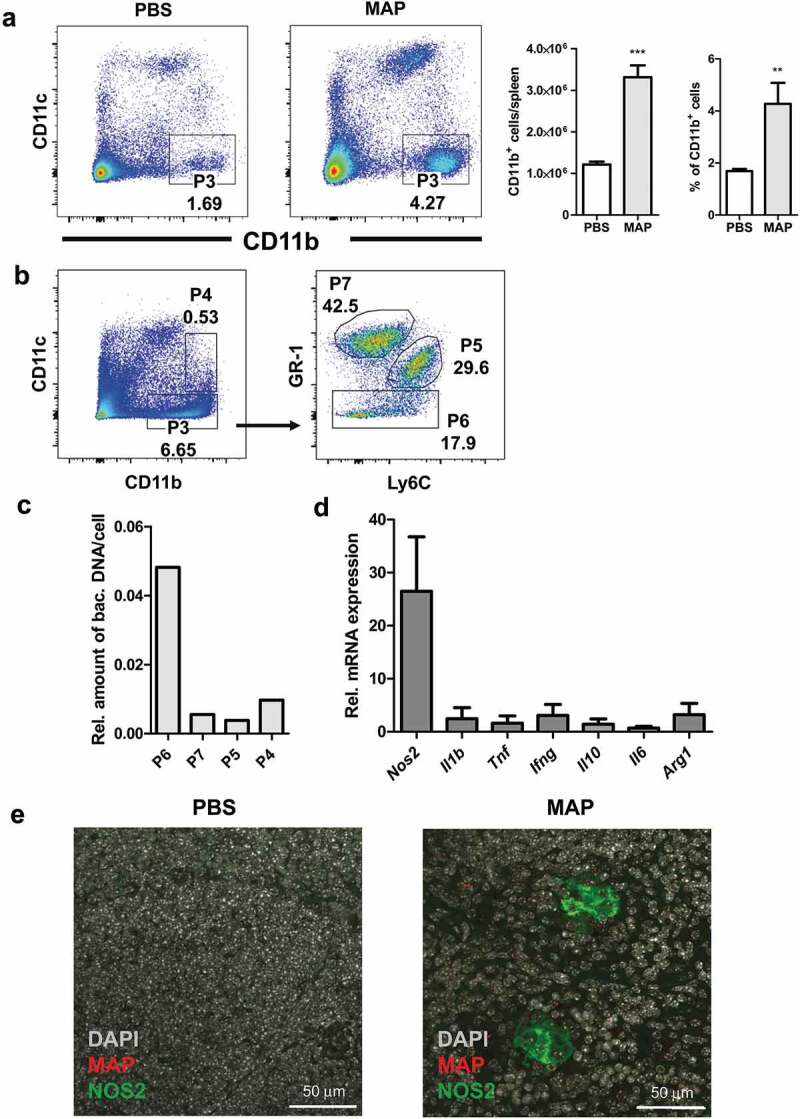
CD11b expressing myeloid cells exhibit enhanced inducible nitric oxide synthase expression after MAP infection.

In infected mice, MAP resided in MAC2 positive myeloid cells. To better understand the role of myeloid cells for MAP persistence, we sorted CD11b^+^CD11c^−^ myeloid cells from the spleens of un-infected control and MAP-infected mice. Infection led to a significant increase of CD11b^+^CD11c^−^ cells (Figure 3(a)). These cells were further separated into three subpopulations (purity ≥ 90%) using Gr-1 and Ly6C markers to yield granulocytes (Gr1^hi^Ly6C^lo^), monocytes (Gr1^int^Ly6C^hi^) and macrophages (Gr1^lo^Ly6C^lo/int^) (Figure 3(b)). Following DNA extraction, qPCR analysis showed that mainly the macrophage fraction was infected with MAP (Figure 3(c)). RT-qPCR also revealed that CD11b^+^CD11c^−^ cells from infected mice expressed markedly high levels of type 2 nitric oxide synthase mRNA (*Nos2*), while expression of *Il1b, Tnf, Il6, Ifng, Il10*, and *Arg1* was only weakly induced (Figure 3(d)).

To determine whether MAP resided in NOS2 expressing macrophages, spleen sections were stained with anti-MAC2, anti-MAP, and anti-NOS2 antibodies. As shown in Figure 3(e), splenic myeloid cells harboring MAP also expressed NOS2. Similar findings were obtained from liver sections of infected mice (Figure S2A). Here, MAP could be shown to reside in NOS2 expressing myeloid cells of granulomas. In agreement, the appearance of NOS2 expressing cells coincided with the formation of clearly defined granuloma containing epithelioid macrophages at 3 weeks of infection (Figure S2B, middle panel). This indicates that NOS2 expression of infected macrophages is delayed and could depend on IFNγ produced by T cells.

### IFNγ and NOS2 contribute to the control of MAP in macrophages

**Figure 4.**
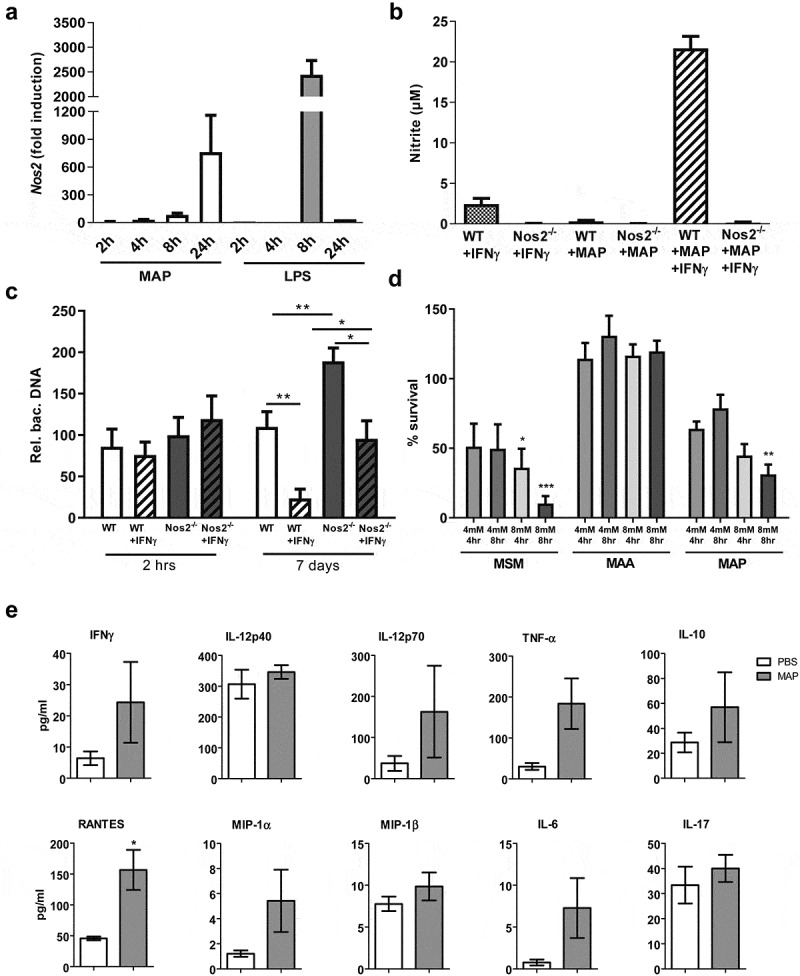
IFNγ and NOS2 contribute to the control of MAP in macrophages.

The almost exclusive presence of MAP in NOS2-expressing cells *in vivo* could be due to a preferential uptake of MAP by NOS2^+^ phagocytes or result from the induction of NOS2 by MAP. We therefore infected bone marrow-derived macrophages (BMDM) *in vitro* with MAP at a multiplicity of infection (MOI) of 10 and analyzed the cells for up to 24 h after infection. Under these conditions, MAP gradually induced *Nos2* mRNA expression over time, whereas LPS that was used as positive control, led to an approximately three-fold higher *Nos2* mRNA expression which already peaked at 8 h (Figure 4(a)). Despite the 600-fold induction of *Nos2* mRNA by MAP alone, significant accumulation of nitrite (as stable end product of NO) was only seen after co-stimulation with MAP and IFNγ (Figure 4(b)).

To investigate the functional relevance of *Nos2* expression in macrophages, wild type (WT) and *Nos2^−/−^* BMDM were infected with MAP *in vitro*. After 7 days, intracellular bacterial load was determined by PCR relative to the 2 h time-point of infection. Whereas in unstimulated WT macrophages the number of bacteria remained largely unaltered, priming of the macrophages with IFNγ 18 h prior to infection resulted in a remarkably decreased bacterial load at day 7 of culture. In the absence of *Nos2*, unstimulated macrophages allowed an approximately twofold increase of the bacterial burden during the observation period while IFNγ treatment slightly reduced the bacterial burden. Importantly, IFNγ-activated *Nos2^−/−^* macrophages harbored significantly less mycobacteria than unstimulated *Nos2^−/−^* macrophages. However, the relative bacterial numbers were higher than in the respective IFNγ-stimulated WT controls (Figure 4(c)). From these data we conclude that macrophages control the growth of MAP by both NOS2-dependent and NOS2-independent mechanisms.

To further demonstrate the role of NO in controlling MAP, we tested whether MAP is directly susceptible to NO. For this we included the *M. avium* strain DSM44156 (MAA), which has been shown to be resistant to NO [27] and *M. smegmatis* strain mc^2^ (MSM) which has been shown to be susceptible for NO [36] as controls. Bacteria suspensions in PBS were treated with the NO donor GSNO (4 and 8 mM) for 4 and 8 h. Survival was monitored by plating und CFU counting. Under these conditions, NO release from GSNO increased over time from approximately 450 µM to approximately 700 µM when bacteria were treated with 4 mM GSNO, while treatment with 8 mM led to a constant exposure of 800 µM over the time (Figure S3). Figure 4(d) shows that survival of MAP and MSM was significantly reduced in the presence of 8 mM GSNO. A tendency of susceptibility was already seen by treatment with 4 mM GSNO. As expected, viability of MAA was not influenced. These data indicate that MAP is at least partially sensitive to the bacteriostatic or bactericidal effects of NO.

Our data indicated that IFNγ enhances NO production in MAP-infected macrophages. To extend these findings to the *in vivo* situation, we determined serum concentration of IFNγ as well as cytokines and chemokines five weeks after infection. As shown in Figure 4(e), in infected mice IFNγ and other immune effectors were upregulated compared to the PBS control, although statistical significance was only reached for RANTES (CCL5) (Figure 4(e) and Figure S4). Nevertheless, local concentrations of such cytokines produced by macrophages and/or T cells might be sufficient to co-trigger the induction of *Nos2* in MAP bearing macrophages.

### NOS2 contributes to the control of MAP infection in mice

**Figure 5.**
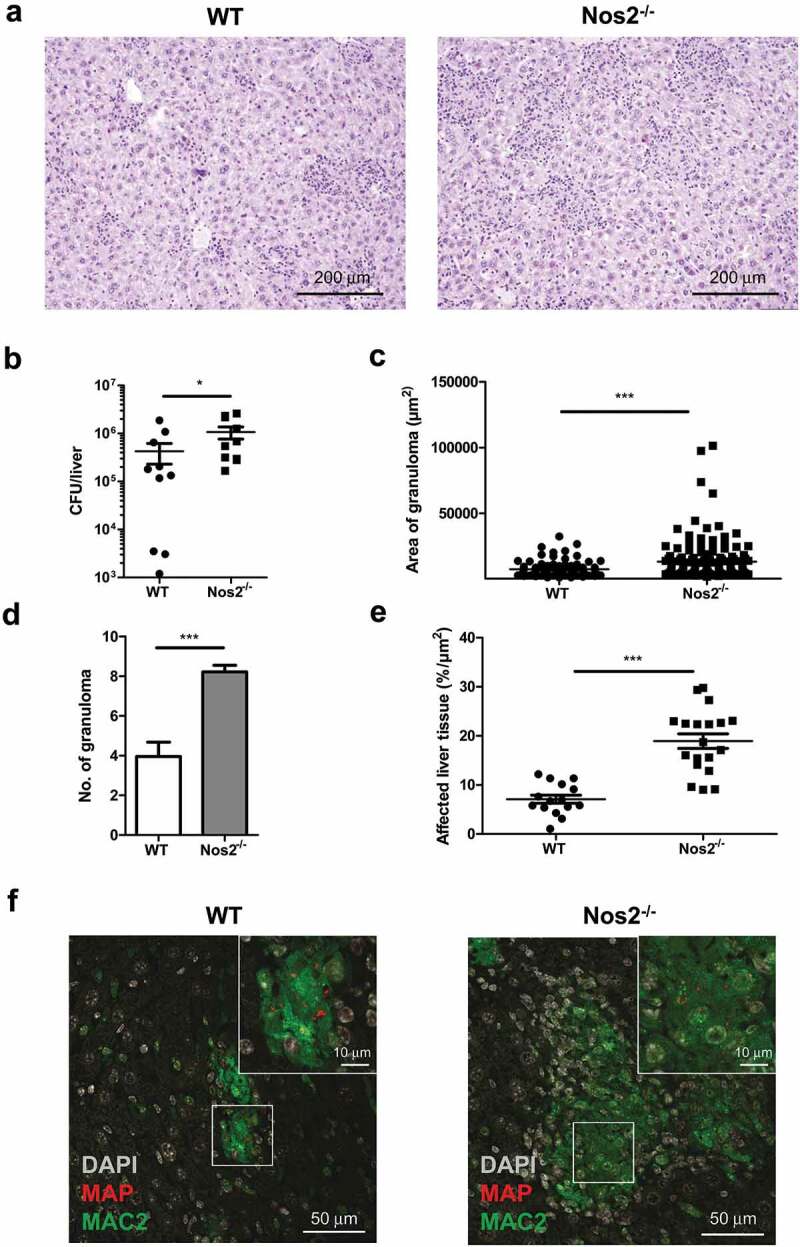
NOS2 contributes to the control of MAP infection in mice.

As shown above, *Nos2* expression by MAP-infected macrophages was associated with reduced bacterial loads *in vitro*. To determine the relevance of this mechanism *in vivo*, we infected *Nos2^−/−^* mice. Interestingly, by 5 weeks post infection *Nos2^−/−^* mice displayed increased spleen weights (Figure S5A) and more severe pathology in liver compared to infected WT mice (Figure 5(a)) indicating more severe inflammation. Correspondingly, a significantly higher bacterial burden was found in the liver of MAP-infected *Nos2^−/−^* mice (Figure 5(b)). However, the gross number of CD4^+^ T cells was unchanged to WT mice (Figure S5B) and only slight differences in serum levels of IFNγ, IL12 and IL6 could be detected (Figure S5C). In contrast, severe multifocal, diffuse granulomatous inflammation, higher numbers of granuloma per section and larger areas of affected liver tissue were observed in infected *Nos2^−/−^* mice compared to WT controls (Figure 5(c–e)). In addition, granuloma in *Nos2^−/−^* mice contained large areas of MAP harboring MAC2 positive macrophages (figure 5(f)). Together, our data indicate that *Nos2* expression and subsequent NO production are involved in the restriction of bacterial growth in mice chronically infected with MAP.

## Discussion

Despite the increasing economic impact of JD, its pathobiology is still unclear. This limits diagnosis, treatment and prevention of disease. The complexity arising from the prolonged process of MAP persistence and replication in the gut leading to pathogenesis and clinical disease is one major difficulty in controlling the pathogen. This particular feature of the disease clearly hampers the use of susceptible animal infection models.

The mouse is commonly used to study virulence and host pathogen interactions of various infectious microorganisms including mycobacteria. Although the mouse model often poorly mimics the complex picture of mycobacterial infection it is able to provide important insights [24,37,38]. Immunocompetent mice are highly resistant to mycobacterial infections. Nevertheless, susceptible inbred strains and genetically modified animals lacking specific components of the immune system allow defined experimentation to understand the role of critical mycobacterial factors or host components involved in pathogenesis [37].

Infection of mice with a high inoculum of 10^8^ CFU via the intraperitoneal route is recommended for reproducible experimental setups [24]. As shown by us and others [34,39–42], under these conditions MAP-infected mice develop chronic granulomatous inflammation in spleen and liver. MAP infection is controlled but not cleared. This is in striking contrast to other *M. avium* subspecies, which have shown to increase in number over the time of infection [27]. Recently we demonstrated that persistence of MAP in mice involves an inadequate IFNβ response [34]. In the present study, we provide evidence that proliferation of MAP in mice is restricted by NOS2/NO activity in infected macrophages in the granulomas. This is remarkably different to infection of NOS2 deficient mice with other *M. avium* subspecies. Here, absence of NOS2/NO improved bacterial clearance and disease [27,43]. To the best of our knowledge, our study is the first reporting effects of NOS2/NO expression on MAP infections *in vivo*.

Infection of mice by MAP elicited specific immune reactions resulting in granulomatous inflammation in spleen and granuloma formation in liver. Increased numbers of cDC and MAP-reactive T cells were observed in the spleens and slightly elevated levels of IFNγ were found in the sera of infected mice. No indication of immune subversion was observed under these conditions. Dendritic cell function and activation of adoptively transferred OVA antigen-specific CD4^+^ T cells was not influenced. In addition, MAP-specific CD4^+^ T cell responses were elicited. Apparently, these immune reactions were able to control the MAP infection but were not sufficient to eliminate the pathogen. This is reminiscent of the situation of MTB infected immunocompetent mice [44,45]. However, it is in striking contrast to mice infected with other *M. avium* subspecies where a cellular immune response develops but is unable to control bacterial growth [27].

The control of mycobacterial infection strongly depends on the types of myeloid cells that are targeted by the pathogens [27]. In the present study, we observed accumulation of mycobacteria containing myeloid cells expressing MAC2 in spleen and liver after seven days of infection. In addition, MAP containing myeloid cells expressing MAC2 were found in the center of granuloma starting at week 3 after infection. However, only myeloid cells in the granuloma centers expressed NOS2. Sorting of splenic myeloid cells revealed that MAP resided in Gr1^lo^Ly6C^lo/int^ macrophages of a CD11b^+^CD11c^-^ cell population expressing *Nos*2.

Our *in vitro* results suggested that IFNγ produced by T cells is necessary for NOS2 expression of MAP containing myeloid cells. In agreement, IFNγ has been shown to be necessary for controlling *M. avium* infection [46]. However, also IFNγ-independent mechanisms of protection do exist as shown by experiments using different inbred mouse strains and IFNγ deficient mice [47,48]. The production of NO by NOS2 apparently restricted MAP growth and disease since the absence of *Nos2* in recombinant mice resulted in a higher bacterial load and increased pathology 5 weeks post infection. In such mice, levels of serum cytokines and chemokines were nearly similar to WT controls. CD4^+^ T cell and serum IFNγ were only marginally increased. Hence, IFNγ alone is most likely not able to control bacterial infection *in vivo* in the absence of NOS2. Although, this emphasizes the *in vivo* importance of CD4^+^ T cells and IFNγ for MAP infection control, it could not be mimicked by our *in vitro* experiments. Here, IFNγ pre-activation of BMDM from Nos2*^−/−^* mice before MAP infection restricted bacterial growth. *In vivo* pathogenic mycobacteria need to infect permissive naïve macrophages to be able to survive in the host. Thereby, they are protected against IFNγ induced killing [49,50]. However, during the course of infection myeloid cells activated by IFNγ or other cytokines most likely are involved in protection against MAP. Hence, to understand the critical role of IFNγ during MAP infection in more detail further studies will be needed.

NO is known to considerably influence infection processes. On the one hand, NO is known for its antimicrobial activity. It might directly restrict bacterial growth and survival. On the other hand, it is an immunomodulatory molecule. NO can either exacerbate tissue damage or limit immunopathology during infections by helping to shut down innate or T cell-driven responses [51–54]. Striking differences are found regarding the mode of action of NO in mice infected with different pathogenic mycobacteria. Experimental infections of *Nos2^−/−^* mice with MTB or *M. bovis* BCG clearly demonstrated a dominant antimicrobial activity of NO. Bacterial numbers and pathology were exacerbated in such mice. In contrast, investigations using virulent MAA revealed that high levels of NOS2/NO expression are induced in inflammatory monocytes [27]. This has detrimental consequences for the host as MAA-infected mice which lack NOS2 show improved pathology and reduced bacterial numbers.

In the present study, we demonstrate that control of MAP infection by NO is most likely due to its direct bactericidal activity. Three weeks after infection, MAP was most prevalently found in macrophages that express NOS2 and thus produce NO. We also show that MAP is sensitive to NO. Hence, although being closely related to other *M. avium* subspecies, the NO-mediated infection control of MAP strongly resembles that of MTB. Despite targeting different organs and hosts, both MAP and MTB can cause latent infections or are completely cleared depending on the immune state of the host and the production of NO [17,55,56].

The presented data help to better understand some aspects of the pathobiology of JD. Whether an animal is capable to mount an early Th1 response, to express NOS2 and to produce NO or not, might be decisive for the development of paucibacillary versus multibacillary JD. Lack of NOS2 expression has been reported in cattle with multibacillary JD [57,58]. While the individual capability to produce NO remains to be demonstrated for MAP-infected animals, *Nos2* gene polymorphism has been shown to correlate with susceptibility to *M. bovis* infection in cattle [59].

It is tempting to speculate on the survival and dissemination strategy of MAP. MAP seems to manipulate the immune system of the host by targeting macrophages and inducing limited production of NO during early stages of infection. This would allow initial replication. Subsequently, increasing NO production due to the action of IFNγ will restrict the expansion of the bacteria. This might avoid early pathology. In addition, MAP is subverting the activation of type I IFN [34]. As type I IFN is a co-inducer of *Nos2*, its strong production would most likely lead to the elimination of the bacteria. Hence, the co-stimulus for the NO-producing enzyme NOS2 is most likely the low amount of serum IFNγ. As result, the bacteria reach a steady state or dormancy. After years, when the infected animals have reached adulthood, bacteria are activated possibly during phases of immunosuppression. Then they multiply and cause pathology. As a consequence, bacteria are shed and are able to infect susceptible newborns of the herd. Thus, MAP manipulates the immune system for its success as pathogen.

In conclusion, our study clearly emphasizes the critical role of NOS2 during MAP infection in mice. Hence *Nos2^−/−^* mice might represent a first step for the development of better infection models with specific knock-out animals to better evaluate MAP pathogenicity and to improve potential treatments in future.

## Supplementary Material

Supplemental Material
