# Supplementary material for: Relevance of inducible nitric oxide synthase for immune control of Mycobacterium avium subspecies paratuberculosis infection in mice
Source: Virulence. 2020 May 14;11(1):465–81. doi: 10.1080/21505594.2020.1763055 (PMC7239028; doi:10.1080/21505594.2020.1763055)
Supplement: Supplemental Material [file KVIR_A_1763055_SM8440.zip › 2020_01_13_suppl_fig_3_4_5.docx]

**Figure S3**

**Figure S4**

**A**

**Figure S5**

**A**

**C**

**B**

**CD4^+^ T cells**
