# Supplementary material for: Relevance of inducible nitric oxide synthase for immune control of Mycobacterium avium subspecies paratuberculosis infection in mice
Source: Virulence. 2020 May 14;11(1):465–81. doi: 10.1080/21505594.2020.1763055 (PMC7239028; doi:10.1080/21505594.2020.1763055)
Supplement: Supplemental Material [file KVIR_A_1763055_SM8440.zip › Supplementary figure legends.docx]

**Supplementary figure legends**

**Figure S1: Related to Figure 1. (A**): Ziehl-Neelsen (ZN) staining showing presence of MAP (shown in red) in liver granuloma five weeks post infection (counterstain malachite green). The boxed region is shown enlarged on the left. (**B**): Liver HE staining showing kinetics of granuloma formation at 1 day, 2 weeks and 3 weeks post infection. Arrows indicates the presence of granuloma.

**Figure S2**: **Related to Figure 3. MAP infection induces NOS2 expression in the liver. (A**): Liver confocal microscopy images showing NOS2 expression (green) among MAP (red) infected cells five weeks after infection. (**B**): **upper panel**: Liver HE staining showing kinetics of granuloma formation at 1 day, 1 week and 3 weeks post infection. **Middle panel**: Liver confocal microscopy images showing kinetics of MAC-2 expressing cells (green) accumulation and presence of MAP (red) in the granuloma Bars indicate 25 µm. **Lower panel**: Confocal microscopy images showing kinetics of NOS2 expression (green) among MAP (red) infected cells over the indicated time points after infection. Bars indicate 25 µm.

**Figure S3: Related to Figure 4. Kinetics of nitrite release from GSNO.** GSNO was used in final concentrations of 4 mM and 8 mM to treat the respective bacterial strains used in **figure 4D.** At the indicated time points, the concentration of nitrite was determined with Griess reagent.

**Figure S4: Related to Figure 4**. Concentrations of cytokines and chemokines in sera from PBS treated (n=4) and MAP-infected (n=5) mice five weeks post infection as determined by multiplex ELISA.

**Figure S5: Related to Figure 5.** (**A**): Spleen weights of WT PBS control (n=3), *Nos2*^-/-^ PBS control (n=4) and MAP-infected WT (n=10) and *Nos2*^-/-^ (n=10) mice. (**B**): Percentage and absolute numbers of CD4^+^ T cells in spleens of WT and *Nos2^-/-^* mice as quantified by flow cytometry. **(C):** Concentrations of cytokines and chemokines in sera from WT and *Nos2^-/-^* mice five weeks post infection as determined by multiplex ELISA. All experiments were performed five weeks post infection. Mean + SEM (t-test * P<0.05).
