# Supplementary figures and images for: Relevance of inducible nitric oxide synthase for immune control of Mycobacterium avium subspecies paratuberculosis infection in mice
Source: Virulence. 2020 May 14;11(1):465–81. doi: 10.1080/21505594.2020.1763055 (PMC7239028; doi:10.1080/21505594.2020.1763055)

**A**


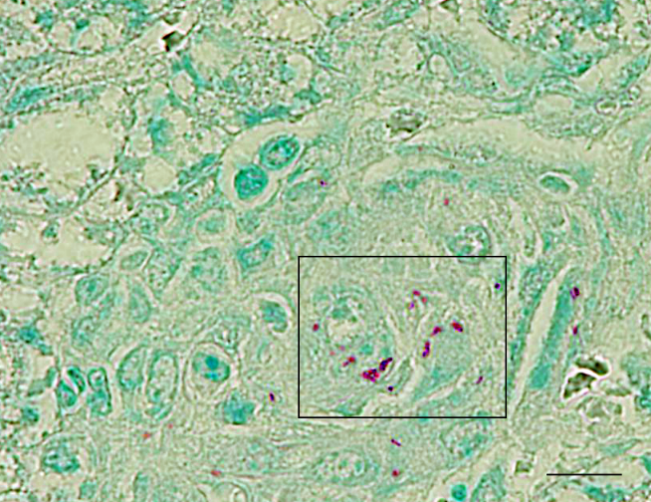


10 μm


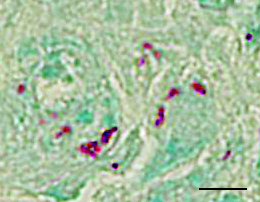


5 μm

**Figure S1**

**1 day**

**2 weeks**

**3 weeks**


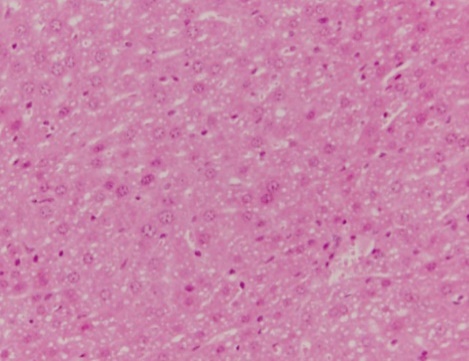

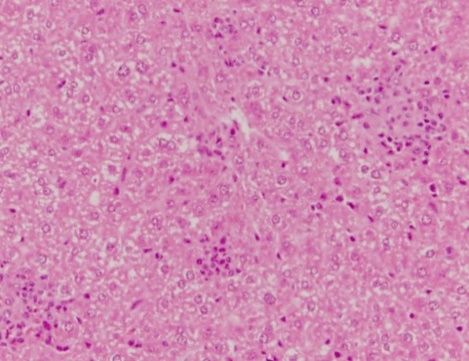

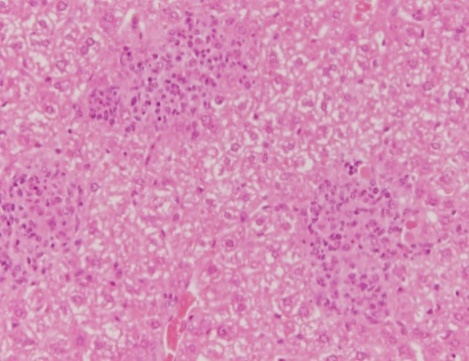


**100 μm**

**100 μm**

**100 μm**

**B**

Supplement: Supplemental Material [file KVIR_A_1763055_SM8440.zip › 2020_01_13_suppl_fig_1.docx]
